# Non-Peptide Agonists and Antagonists of the Prokineticin Receptors

**DOI:** 10.3390/cimb44120431

**Published:** 2022-12-12

**Authors:** Roberta Lattanzi, Rossella Miele

**Affiliations:** 1 Department of Physiology and Pharmacology “Vittorio Erspamer”, Sapienza University of Rome, Piazzale Aldo Moro 5, I-00185 Rome, Italy; 2 Department of Biochemical Sciences “A. Rossi Fanelli”, CNR-Institute of Molecular Biology and Pathology, Sapienza University of Rome, Piazzale Aldo Moro 5, I-00185 Rome, Italy

**Keywords:** prokineticis, prokineticin receptors, orthosteric and allosteric ligands, triazine-compounds, PKRs agonists, PKRs antagonists

## Abstract

The prokineticin family comprises a group of secreted peptides that can be classified as chemokines based on their structural features and chemotactic and immunomodulatory functions. Prokineticins (PKs) bind with high affinity to two G protein-coupled receptors (GPCRs). Prokineticin receptor 1 (PKR1) and prokineticin receptor 2 (PKR2) are involved in a variety of physiological functions such as angiogenesis and neurogenesis, hematopoiesis, the control of hypothalamic hormone secretion, the regulation of circadian rhythm and the modulation of complex behaviors such as feeding and drinking. Dysregulation of the system leads to an inflammatory process that is the substrate for many pathological conditions such as cancer, pain, neuroinflammation and neurodegenerative diseases such as Alzheimer’s and Parkinson’s disease. The use of PKR’s antagonists reduces PK2/PKRs upregulation triggered by various inflammatory processes, suggesting that a pharmacological blockade of PKRs may be a successful strategy to treat inflammatory/neuroinflammatory diseases, at least in rodents. Under certain circumstances, the PK system exhibits protective/neuroprotective effects, so PKR agonists have also been developed to modulate the prokineticin system.

## 1. Introduction

The prokineticin family comprises a group of secreted peptides found in all species, from invertebrates to mammals. In 1999, the first member of the family was isolated from the skin secretion of the frog Bombina variegata and named Bv8 [1]. Subsequently, homologs of Bv8 were identified in lizards, in fish, in the venom of the black mamba (MIT, Mamba Intestinal Toxin) [2,3] and also in mammals. The mammalian proteins, encoded by two different genes, are named prokineticin 1 (PK1) and prokineticin 2 (PK2) for their ability to contract the guinea pig ileum. PK1, the ortholog of MIT, is also called EG-VEGF (endocrine gland-derived vascular endothelial growth factor) because of its ability to induce proliferation, migration and fenestration in the endothelial cells of steroid-synthesizing glands [4,5], while PK2, the ortholog of Bv8, is also called mammalian Bv8 (mBv8). All proteins share a common structure characterized by a conserved N-terminal sequence consisting of alanine, valine, isoleucine, triptophan, glicin, and alanine. For this reason, they are also called AVITGA proteins [6,7]. PKs are also characterized by 10 cysteine residues forming five disulfide bridges and a tryptophan residue at position 24, which is crucial for receptor binding. Prokineticins bind with high affinity to two G protein-coupled receptors (GPCRs), prokineticin receptor 1 (PKR1) and prokineticin receptor 2 (PKR2), which have 85% amino acid identity and differ mainly in their N-terminal region [8]. PKRs are coupled to Gq, Gi and Gs, and activate different intracellular signaling pathways depending on the cell type [6]. Moreover, PKR2 has been shown to undergo dimerization in human neutrophils [9,10]. Both receptors are widely expressed in various organs and tissues as well as in the peripheral and central nervous system, with PKR2 being more highly expressed than PKR1 [6].

Prokineticins can be classified as chemokines based on their structural features, such as their small size, presence of cysteine residues, ability to bind to GPCRs and chemotactic and immunomodulatory functions [11]. Intensive research in recent years has shown that the prokineticin system is involved in a variety of physiological functions such as angiogenesis and neurogenesis [12,13,14], hematopoiesis, control of hypothalamic hormone secretion [15], regulation of circadian rhythm [16,17] and modulation of complex behaviors such as food intake and drinking [18,19,20]. Dysregulation of the system leads to an inflammatory process that is the substrate for many pathological conditions such as cancer, pain, neuroinflammation and neurodegenerative diseases such as Alzheimer’s and Parkinson’s disease [12,21,22,23]. The use of nonpeptide PKR’s antagonists reduces PK2/PKRs upregulation triggered by various inflammatory insults, at least in rodents, suggesting that pharmacological blockade of PKRs may be a successful strategy for treating inflammatory/neuroinflammatory diseases.

PKR’s agonists have also been developed to modulate the prokineticin system.

## 2. Receptor-Prokineticin Interactions

The characteristic architecture of all chemokines consists of a disordered sequence called the “signal domain” followed by a structured “core domain” consisting of a three-stranded β-sheet and a C-terminal helix. The final structure, after the formation of a head-tail dimer, consists of a six-stranded β-sheet platform topped by two α-helices [24]. Similarly, NMR analysis shows that MIT [25] and Bv8 [26] adopt a similar structure, a colipase fold structure characterized by a compact core domain consisting of two antiparallel three-stranded β-sheets and a prominent amino-terminal sequence that plays a key role in biological activity.

The amino-terminal AVITGA sequence is evolutionarily highly conserved and occurs in mammalian PK1 [8] and in all described PK2 splice variants [6,27,28]. The importance of the N-terminal residues is supported by the observation that the addition or replacement of only a single amino acid results in PK1 mutants that possess strong antagonistic activity [29], whereas deletion of the N-terminal alanine residue of Bv8 results in a significant, but not complete, loss of biological activity [30].

In addition, the missense mutation described in patients with Kallmann syndrome (KS), which results in a substitution of glycine in the N-terminal AVITGA sequence, determines inactivation of the protein with a subsequent loss of function [31,32]. These data are consistent with several studies on many other chemokines.

Similarly, the C-terminal domain has an important functional role. Indeed, proteins with mutations of cysteine residues in the C-terminus or complete deletion of the carboxyl domain are not biologically active [29]. The hydrophobic tryptophan residue at position 24 (Trp24) exposed on the protein surface is essential for receptor binding and activation. Depending on the dose used, the mutant obtained by replacing Trp24 with alanine behaved like a PK receptor agonist or prevented Bv8-mediated hypergesia [33,34].

Chemokine receptors bind ligands via a mechanism called the two-sided model, which involves two phases: a first step through the interaction of the chemokine core domain with the N-terminus and extracellular loop 2 (ECL2) of the receptor (site 1) and a second step consisting of the insertion of the flexible N-terminal chemokine signal sequence into the orthosteric pocket of the GPCR (site 2), triggering conformational changes that initiate intracellular signal transduction [35].

PKR1 and PKR2 site 1 have been characterized, demonstrating their importance for binding specificity. The sequence divergence between PKR1 and PKR2 receptors, located almost exclusively in the N-terminal region, enables ligand selection at this stage [36]. ECL2 also contains important residues for binding such as asparagine (Q) 210 and tryptophan (W) 212. Functional analysis of the PKR2 KS mutant due to the substitution of Q210 by arginine (Q210R) shows no effect on protein expression, but strongly affects the ability to bind ligands [37]. Similarly, in vivo incorporation of a photoactivatable unnatural amino acid p-benzoyl-L-phenylalanine using amber codon suppression technology demonstrated the critical role of W 212 in ligand binding [38].

The orthosteric prokineticin receptor binding site TM (site 2) interacts with the prokineticin amino sequence AVITGA and facilitates a conformational change required for receptor activation. The importance of this interaction is evidenced by the fact that the PKR2 splice variant TM4-7 can be activated by ligands even in the absence of the regions comprising the transmembrane, TM1, TM2 and TM, although site 1 was absent [39]. Similarly, unnatural small molecule ligands identified as prokineticin receptor agonists and antagonists bind exclusively to PKR’s TM bundling site [36,40].

**Figure 1:**
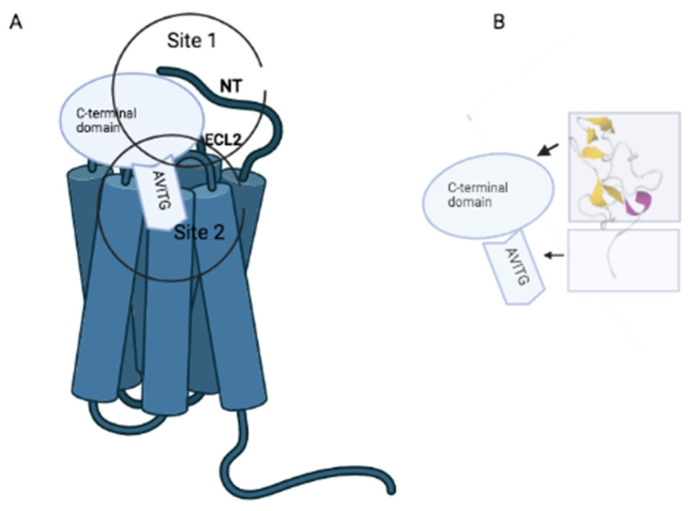
(**A**). Proposed mechanism for prokineticin-receptor interaction. Prokineticins bind PKR receptors via two stages: a first step through the interaction of the C-terminal prokineticin domain with the N-terminus (NT) and extracellular loop 2 (ECL2) of the receptor (page 1) and a second step consisting of the insertion of the AVITGA sequence into the orthosteric pocket of PKR (page 2). (**B**). Three-dimensional structure of Bv8 (Data Bank: PDB ID: 2kra) and schematic showing regions corresponding to the C-terminal domain and AVITGA.

Homology modeling studies have described a TM bundling site of PKR that identifies crucial residues for interaction with ligands. These same residues were highlighted as important because of their specific spatial location in the TM-binding site in receptors as diverse as Arg1443.32 of PKR1, which is analogous to Asp1133.32 of the β2-adrenergic receptor, which is an experimentally well-established site of receptor interaction with both agonists and antagonists. In addition, Arg3076.58, which interacts with endogenous ligands and non-peptidic small molecule ligands, is also present in other peptidic GPCRs such as the GnRH receptor, the NK2 tachykinin receptor, the AT1A angiotensin receptor and the CXCR1 chemokine receptor. In particular, the crystallographic X-ray structure of the CXCR4 chemokine receptor evidences a specific interaction between position 6.58 and a cyclic peptide antagonist. Finally, glutamic acid in position 2.61 of the hPKR was found to be essential for the binding of the antagonist by electrostatic interaction with a specific positive charge of the ligand [36] (Figure 1).

## 3. Non-Peptide Orthosteric Ligands of Prokineticin Receptors

GPCRs have a common binding pocket for small molecules in the TM cavity that can accommodate natural and nonpeptidic orthosteric ligands. Several compounds have been identified as orthosteric ligands for prokineticin receptors and tested on various biological systems (Table 1).

In recent years, a series of triazine-guanidine compounds (from PC1 to PC36) capable of binding in the pocket of GPCRs have been synthesized [41]. These molecules have a triazine-guanidine group mimicking the N-terminal sequence of AVITGA and a methoxybenzyl aligned as a tryptophan residue at position 24 [8,33,34]. They all bind the PKRs between TMs 3, 4, 5, 6 and 7 with high affinity as shown by radioligand binding assays and BRET (bioluminescence resonance energy transfer) experiments [42]. Almost all of them show the same affinity for PKR1 and PKR2, with the exception of PC25, which is 300 times more selective for PKR1 than for PKR2 [42]. In in vivo experiments, they all act as antagonists by completely abolishing Bv8-induced hyperalgesia in mice [43].

Nevertheless, PC1 is the best characterized. In vitro, it inhibits the Bv8-induced release of calcium currents in CHO cells, and in vivo, it is able to reverse many pathological effects due to PK2 overexpression. PC1 actually reduces inflammatory pain and edema by decreasing the upregulation of PK2 in inflamed tissue [44]. In mice, it also abolishes neuropathic pain caused by sciatic nerve ligation [45,46,47], diabetes [48] or chemotherapeutic agents [49,50,51]. PC1 is able to reverse deficits and improve spatial memory in a rat model of Alzheimer’s disease by reducing neuronal death triggered by upregulation of PK2 [52,53,54]. In addition, both PC1 and PC7 reduce demyelination in Experimental Autoimmune Encephalomyelitis (EAE), a mouse model of multiple sclerosis [55], and PC7 could be used as a drug in preeclampsia because reducing PK1 overexpression improves pregnancy outcome [56,57]. In contrast, in primary murine cell cultures or in rat organotypic hippocampal slices, PC7 shows a neurotoxic effect increasing neuronal damage by oxygen-glucose deprivation (OGD) [58].

PKRA7 [(3R)-1-(4-fluoro-3-methoxybenzyl)-N-(9-chloro-3,4-dihydro-2H-1,5-benzodioxepin-7-ylmethyl)-N-isobutylpyrrolidine-3-carboxamide] was synthesized to mimic mutant peptides obtained by replacing the alanine residue with methionine and adding methionine at the N-terminus of PK2. It inhibits prokineticin receptors with IC50 values of 5.0 and 8.2 nM for PKR1 and PKR2, respectively. PKRA7 penetrates the blood-brain barrier and inhibits angiogenesis and macrophage infiltration in mice transplanted with glioblastoma and pancreatic cancer [59]. PKRA7, which binds to PKR1 and PKR2, significantly reduces the severity of symptoms in mice with collagen-induced arthritis by inhibiting macrophage infiltration in the synovial membrane and the production of the inflammatory cytokines IL -1, IL -6, TNF-α [60]. Recently, PKRA7 was also shown to play a role in reducing the PK2-mediated inflammatory process during uropathogenic *Escherichia coli* (UPEC)-induced orchitis by suppressing PK2 activity [61].

In rats with cyclophosphamide- (CYP -) induced cystitis, PK2 and PKR1 are upregulated to different degrees. Blocking PKRs with PKRA7 showed no effect on micturition reflex activity and bladder sensation in control rats, whereas it increased the voiding volume, prolonged the voiding interval, and improved visceral hyperalgesia in rats with CYP -induced cystitis. In conclusion, the PK2/PKR1 pathway contributes to the modulation of inflammation-induced voiding dysfunction and spontaneous visceral pain [62].

PKRA7 also abolishes the effect of metformin (Met) in mice with streptozotocin-induced diabetes. Diabetic mice exhibit metabolic abnormalities, abnormal myocardial levels, and marked apoptosis associated with the downregulation of PK2 and PKR expressions. In these mice, H9c2 cardiac cells exposed to high glucose (HG) showed decreased PK2/PKR expression and decreased p-AKT/AKT and p-GSK3β/GSK3β ratios, and these effects were abolished by Met. However, treatment with PKRA7 or an AKT inhibitor abolished the Met-induced effects on cardiomyocytes exposed to HG. These results suggest that Met can activate the PK2/PKR-mediated AKT/GSK3β signaling pathway, thereby improving cardiac function and attenuating apoptosis in diabetic mice [63].

Recent evidence supports the essential role of trehalose (TLS) in cardiomyocyte survival signaling. The mechanisms of the protective effect of trehalose (TLS) in the streptozotocin-induced diabetic model were analyzed (DM). Cardiomyocytes exposed to high glucose (HG) were treated with TLS in the absence or presence of the PK2 antagonist PKRA7. Treatment with TLS reversed the diabetic phenotype only in the absence of PKRA7. These results demonstrate that TLS rescues DM-induced myocardial function via the PK2/PKR pathway [64].

Endocrine gland-derived vascular endothelial growth factor (EG-VEGF) is highly expressed in the human placenta; it contributes to placental vascularization and growth, and its abnormal expression has been associated with pregnancy pathologies such as preeclampsia and fetal growth restriction. PKRA7 and PC7 were tested independently or in combination in trophoblast cells and during early gestation in gravid mice to reveal the endogenous functions of EG-VEGF. The results provide evidence for the potentially safe use of PC7 or PKRA7 to improve pregnancy outcomes by promoting fetoplacental growth in health and for the potential therapy of pregnancy pathologies [56].

In mouse models of Parkinson’s disease (PD), both the MPTP and MitoPark transgenic mouse models, PK2 levels are markedly elevated in the striatum during the first phase of neuronal degeneration before the onset of motor symptoms. In contrast, PK2 levels are very low in healthy mice. In dopaminergic neurons, PK2 reduces MPTP-induced neuronal cell death and oxidative stress, and promotes mitochondrial biogenesis and survival by upregulating PGC-1 and TFAM and activating ERK and AKT signaling pathways 

All biochemical and behavioral deficits induced by MTPT in mice are positively altered by intrastriatal PK2 treatment, and the administration of the PKRs antagonist PKRA7 potentiates the deleterious effects induced by MTPT [65,66].

**Table 1:** Non peptide orthosteric ligands of prokineticin receptors.

Drug	Biological System	Effect	References
PC1	CHO cells	Inhibition of Bv8-induced intracellular Ca^2+^ mobilization	[41]
	Primary cortical cultures	Inhibition of Aβ_1-42_ induced neuronal death; Reduction of PK2-induced increase of AMPA currents	[52,53]
	Tg2575 hippocampal slides	Rescue of LTP impairment	[52]
	Inflamed mice	Reduction of CFA-induced inflammatory pain	[44]
	Mice with neuropathy induced by nerve ligation	Reduction of CCI and SNI-induced neuropathic pain	[45,46,47]
	Mice with neuropathy induced by chemotherapeutics	Reduction of bortezomid and vincristine -induced neuropathic pain	[49,50,51]
	Mice with diabetic neuropathy	Reduction of streptozotocin -induced neuropathic pain	[48]
	Rats with intracerebroventricular infusion of Aβ_1-42_	Reduction of Aβ_1-42_ -induced cognitive impairment	[54]
PC1 and PC7	Mice with autoimmune encephalomyelitis (EAE)	Efficacy in reducing EAE severity	[55]
PC7	Primary murine cell cultures or rat organotypic hippocampal slices	Increasing of neuronal damage induced by oxygen-glucose deprivation (OGD)	[58]
PC7	Porcine primary trophoblast cells	Abolishing of PK1-induced expressions of genes involved in angiogenesis	[57]
PKRA7	Glioblastoma and pancreatic cancer	Inhibition of angiogenesis and macrophage infiltration	[59]
	Arthritis	Inhibition of macrophage infiltration and production of inflammatory cytokines	[60]
	Cystitis	Modulation of inflammation-mediated voiding dysfunction and spontaneous visceral pain	[62]
	Orchitis	Inhibition inflammatory process	[61]
	Diabetic Cardimyocites	Inhibition of metformin effect	[63]
		Inhibition of trehalose effect	[64]
	Pregnancy pathologies	Induction of pregnancy outcome enhancing feto-placental growth	[56]
	Parkinson’s Disease	Increase of the effects induced by MTPT	[65]

## 4. Non-Peptides Allosteric Ligands of Prokineticin Receptors

Allostery is a common biological phenomenon that describes the ability of a small molecule modulator to interact with the receptor at a site other than orthosteric binding and to activate the receptor even in the absence of an orthosteric agonist. Allosteric agents are thought to act by stabilizing a particular conformation of the GPCR by affecting the equilibrium between pre-existing conformational states of the GPCR.

In silico screening identified a selective PKR1 agonist, IS20, that binds the potential allosteric PKR1 site in the transmembrane domain through interactions between the helices III, V, VI, and VII and with the helix II, which is only marginally involved.

In the cardiovascular system, activation of PKR1 has beneficial effects on vascular formation and cell survival [40]. In vivo, the specific binding of IS20 to PKR1 has been shown to protect cardiac function and improve survival in mouse models of myocardial infarction [67]. In models of doxorubicin-induced cardiotoxicity, systemic activation of PKR1 by intraperitoneal injections of IS20 significantly protected cardiac cells by preventing the decline in diastolic ejection fraction observed in vehicle-treated controls. A survival analysis impressively demonstrated that systemic treatment with IS20 reduced doxorubicin-induced mortality without altering the cytotoxic and anticancer effects of DOX in breast cancer lines or in mouse breast cancer models [68]. These data therefore pave the way for the development of PKR1 agonists to prevent cardiovascular complications in cancer.

## 5. Conclusions

**Figure 2:**
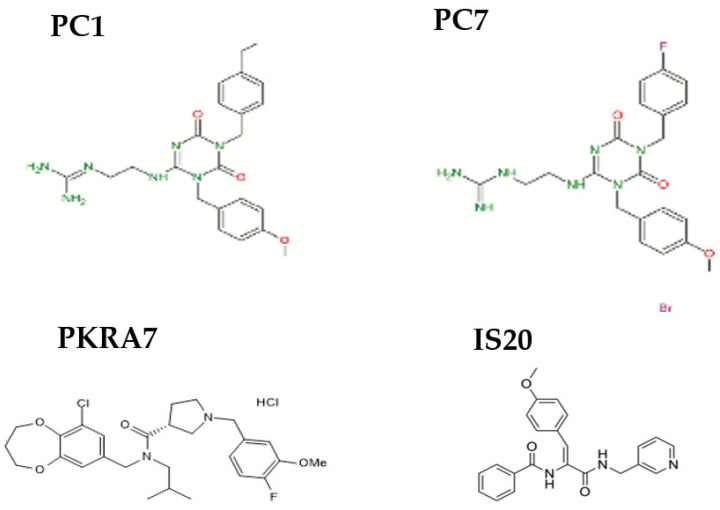
Chemical structures of non-peptide orthosteric and allosteric ligands of prokineticin receptors.

The prokineticin system, which includes the ligands PK1 and PK2 and the two receptors PKR1 and PKR2, is widely distributed in the peripheral and central nervous system and in many peripheral organs and tissues of mammals. It is involved in numerous physiological processes, but deregulation of the system can lead to many pathological conditions such as chronic pain, inflammation and neuroinflammation with neurotoxic or neuroprotective properties. Therefore, the use of PKR agonists or antagonists seems to be essential to maintain or enhance the protective effect of the PK system or to reduce its deleterious effect (Figure 2).

In conclusion, the prokineticin system represents a new potential pharmacological target for the development of innovative therapies.

## Data Availability

Not applicable.
